# Hepaticocystic Duct in an Era of Laparoscopic Cholecystectomy

**DOI:** 10.1055/s-0041-1731428

**Published:** 2021-07-19

**Authors:** Jignesh A. Gandhi, Pravin Shinde, Sadashiv N. Chaudhari, Amay Banker

**Affiliations:** 1 Department of General Surgery, Seth G. S. Medical College and KEM Hospital, Mumbai, Maharashtra, India

**Keywords:** hepaticocystic duct, extrahepatic biliary tree, agenesis of common bile duct

## Abstract

The biliary tract is notorious for its variable anatomy. A persistent hepaticocystic duct with agenesis of common bile duct is a rare biliary anomaly that creates a diagnostic dilemma and can add to the operative difficulties. It is important to diagnose this anomaly preoperatively since the gallbladder forms an integral part of bilioenteric continuity and an inadvertent cholecystectomy can lead to a surgical catastrophe. If diagnosed, surgeons can plan definitive treatment in the form of biliary diversion. We present a case of a 22-year-old man, who presented to us with obstructive jaundice and cholangitis. The biliary system was decompressed initially with a percutaneous transhepatic biliary drainage and an endoscopic retrograde cholangiogram established the diagnosis of a type IV hepaticocystic duct preoperatively in our case. Since diagnosis was made prior to operative intervention, we were able to perform a cholecystojejunostomy to maintain biliary continuity. The patient was discharged with an uneventful postoperative course. To our knowledge, this is the first report of such a variation being diagnosed preoperatively. We are also presenting a brief review of literature about persistent hepaticocystic ducts and the embryological basis of their origin.

The biliary tract is well known for variations in its anatomy. Failure to identify these variations may lead to intraoperative catastrophes. A hepaticocystic duct is one such rare anomaly wherein the right and left hepatic ducts are found to be draining into the gallbladder with agenesis of the common hepatic and common bile ducts (CBDs). 1 Further drainage of the bile from the gallbladder to the duodenum is via a long cystic duct. Few cases of hepaticocystic duct have been reported in literature so far and this is the only case diagnosed preoperatively. 1 2 3 4 5 6 7 8 9 10 11 Preoperative diagnosis helps plan definitive biliary diversion and also gives the option of using the gallbladder to establish bilioenteric continuity.

## Case Report

A 22-year-old man was admitted with chief complaints of recurrent episodes of pain in abdomen and fever with chills for past 2 days. He gave a history of two episodes of jaundice in the past which were managed conservatively.

The patient was febrile, tachycardic, and icteric on admission. Physical examination revealed tenderness in right hypochondrium. Laboratory data revealed a leucocytosis (16,000/mL) with elevated levels of total bilirubin (20 mg/dL) and direct bilirubin (18 mg/dL).

**Fig. 1:**
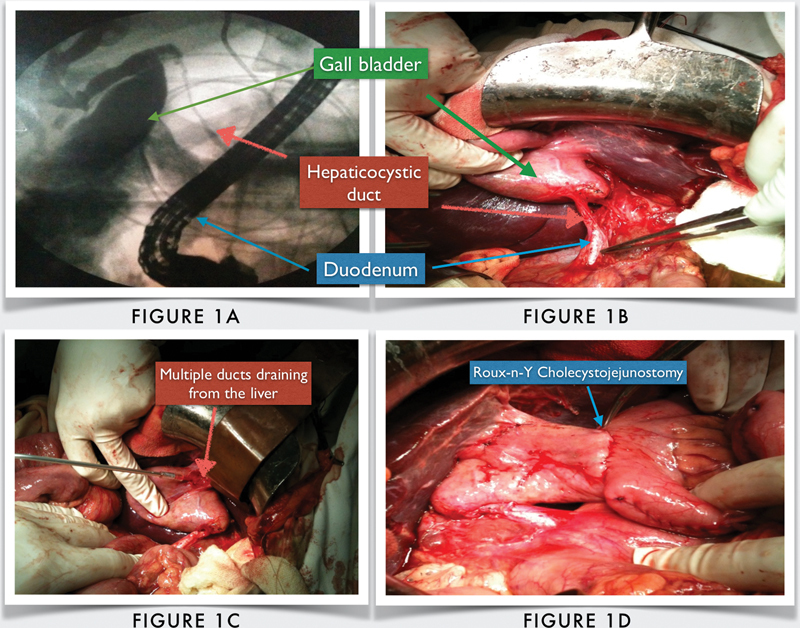
( **A** ) Endoscopic retrograde cholangiogram picture depicting the hepaticocystic duct. ( **B** ) Intraoperative picture showing the same anatomy. ( **C** ) Multiple biliary ducts draining into the gallbladder seen on opening the gallbladder. ( **D** ) Roux-en-Y cholecystojejunostomy.

A contrast enhanced computed tomography of abdomen showed a mass near the hilum with dilated intrahepatic biliary radicals. Provisional diagnosis of obstructive jaundice with cholangitis was made and percutaneous transhepatic biliary drainage (PTBD) was planned to decompress the biliary system. As a cholangiogram at the time of PTBD was unable to delineate the complex biliary anatomy in this patient, an endoscopic retrograde cholangiogram (ERC) was performed. ERC revealed multiple small ducts draining into the gallbladder with absent right and left hepatic, common hepatic, and CBDs ( Fig. 1A ). The gallbladder was found to be draining into the duodenum by a long thin cystic duct.

On elective exploration, multiple small hepatic ducts were seen entering the gallbladder which drained into the duodenum via a cystic duct ( Fig. 1B, C ). Diagnosis of a type IV hepaticocystic duct was confirmed and a Roux-en-Y cholecystojejunostomy was performed ( Fig. 1D ). The postoperative course of the patient was uneventful, and he was discharged on the 10th postoperative day.

On follow-up after 1 month, he was nonicteric with a repeat percutaneous transhepatic cholangiogram demonstrating a free flow of bile from liver into gallbladder and then into the jejunum. At 6-month follow-up after the surgery, the patient was asymptomatic.

## Discussion

In 1958, Braasch categorized all known biliary tract anomalies with a hepaticocystic duct being the rarest type. 12 Losanoff et al were the first to describe this anomaly wherein there was agenesis of the CBD and the hepatic ducts directly drained into the gallbladder. 1 Final biliary drainage into the duodenum was via a long and narrow cystic duct. Losanoff et al termed this anomalous extrahepatic biliary tree as the “hepaticocystic duct.” Other authors have also termed this condition as “transverse lie of gallbladder,” “interposition of gallbladder,” or “cholecystohepatic duct.” 1 3 4 5 In agreement to Losanoff et al, since the flow of bile is from the hepatic ducts to the cystic duct, we preferred the term “hepaticocystic duct” for this condition. 9

**Fig. 2:**
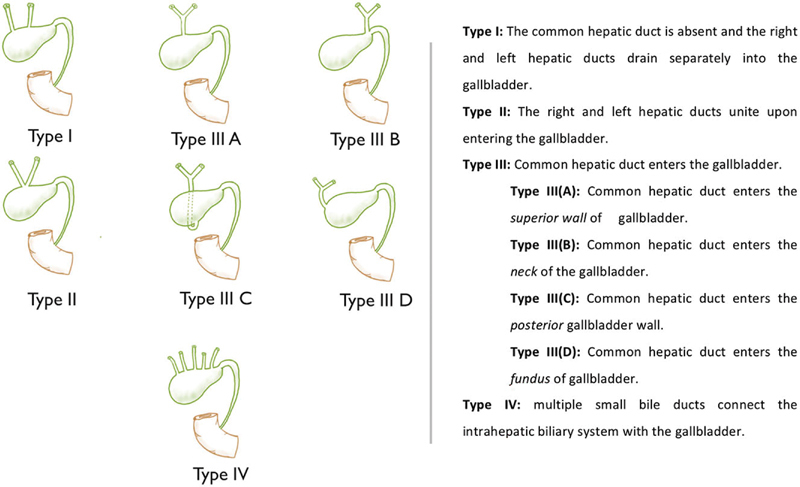
Types of hepaticocystic duct.

Knowledge of the embryological development of the biliary system is necessary to understand the hepaticocystic duct. At fourth week of gestation, the hepatic diverticulum arises ventrally from the primitive endoderm. At the time of appearance of the cystic diverticulum, proliferation of the cells at the junction of hepatic and cystic ducts forms the CBD. 8 It is postulated that a failure of recanalization of the CBD with persistence of fetal communication between gallbladder and liver results into a hepaticocystic duct wherein the left and right hepatic ducts drain into the gallbladder directly with agenesis of the CBD. 13 The cystic duct remains the final pathway of drainage of bile into the duodenum. Losanoff et al classified the hepaticocystic duct into four types ( Fig. 2 ). 9

Cholelithiasis and other gallbladder diseases have been described in most patients with a hepaticocystic duct and such patients may undergo biliary surgery with a high risk of iatrogenic injury. In most of the cases described previously, diagnosis was made intraoperatively at the time of cholecystectomy. 7 14 A preoperative ultrasound may raise suspicion about an anomalous hepatic duct–gallbladder junction. Cholangiographic confirmation of the biliary anatomy prior to cholecystectomy can help prevent intraoperative difficulties, and guide decision-making when such a complex biliary anatomy exists. We were unable to confirm the diagnosis even on a cholangiogram at the time of PTBD, and the unusual biliary anatomy prompted us to perform an ERC prior to surgery. We advocate the use of ERC in cases where biliary anatomy is not adequately delineated, to rule out this condition prior to any operative intervention.

Our patient had type IV anomaly and the importance in identifying this particular type preoperatively is that the gallbladder forms an important part of bilioenteric continuity. Failure of initial recognition of the anomaly may be followed by removal of the gallbladder inevitably resulting in discontinuity of biliary drainage leading to disastrous consequences.

Since we were able to diagnose the condition preoperatively, we favored a cholecystojejunostomy which provided an adequate drainage of the biliary system. It is also important to differentiate this condition from the accessory ducts of Luschka, or the more appropriately named “subvesical” ducts where there is an aberrant drainage of the biliary system into the gallbladder *without* agenesis of the CBD. 15 A cholecystectomy, although difficult, is possible in cases with a duct of Luschka, whereas it would be a surgical catastrophe in cases of a type IV hepaticocystic duct. In this type of anomaly, a hepaticojejunostomy is also not viable due to multiple small ducts draining into the gallbladder. In all other types of hepaticocystic duct, a gallbladder resection may be performed but biliary continuity has to be established with a Roux-en-Y hepaticojejunostomy. 7

## Conclusion

A persistent hepaticocystic duct is one of the rarest anomalies of the biliary system. The potential of iatrogenic injury and the consequences that follow is high if the absence of CBD remains undiagnosed. Preoperative cholangiographic confirmation of the complex biliary anatomy can help in planning the appropriate surgical procedure such as a cholecystojejunostomy or a Roux-en-Y hepaticojejunostomy.
